# Evaluation of point of care tests for the diagnosis of cutaneous leishmaniasis in Suriname

**DOI:** 10.1186/s12879-018-3634-3

**Published:** 2019-01-07

**Authors:** Henk D. F. H. Schallig, Ricardo V. P. Hu, Alida D. Kent, Merlin van Loenen, Sandra Menting, Albert Picado, Zippora Oosterling, Israel Cruz

**Affiliations:** 1 Academic Medical Centre, Department of Medical Microbiology, Parasitology Unit, Meibergdreef 9, 1105 AZ Amsterdam, The Netherlands; 2 Dermatology Service, Ministry of Health, Tourtonnelaan 5, Paramaribo, Suriname; 3 Department of Parasitology, Anton de Kom University, Kernkampweg, Paramaribo, Suriname; 4 Foundation for Innovative New Diagnostics, Geneva, Switzerland

**Keywords:** Cutaneous leishmaniasis, Diagnostics

## Abstract

**Background:**

Cutaneous leishmaniasis (CL) is a serious health problem in Suriname. To expand the diagnostic options, two newly developed diagnostic tests, i.e. the rapid diagnostic test CL Detect™ Rapid Test (CL Detect) and the Loopamp™ *Leishmania* Detection Kit (Loopamp) were evaluated.

**Methods:**

Diagnostic test performance was compared to the routine diagnostic approach in place, i.e. clinical symptoms combined with microscopy, and to polymerase chain reaction (PCR), which was used as a reference standard. The study population (*n* = 93) was a typical representation of the CL affected population in Suriname and mainly infected with *Leishmania guyanensis*.

**Results:**

CL Detect had a very low sensitivity compared to microscopy (36.7%) or PCR (35.8%), due to a high number of false negative results. The specificity of the CL Detect compared to microscopy and PCR was 85.7 and 83.3% respectively. Loopamp sensitivity was 84.8% compared to microscopy and 91.4% compared to PCR. The Loopamp test had a moderate specificity (42.9%) compared to microscopy, but a good specificity compared to PCR (91.7%).

**Conclusion:**

The CL Detect is not likely to be a good replacement for the routine diagnostic procedure for CL in Suriname. The high sensitivity of the easy to perform Loopamp enables the implementation of sensitive molecular diagnosis in resource limited settings.

## Keypoints

A new rapid diagnostic test for the parasitic skin disease, cutaneous leishmaniasis, did not perform well in a diagnostic evaluation in Suriname and it cannot replace the standard diagnostic procedures in place, which is microscopy. A new molecular diagnostic test may have the potential to enable molecular diagnosis of CL in less resourced settings.

## Background

Leishmaniasis is a complex of disease caused by parasitic kinetoplastid flagellates of the genus *Leishmania* and manifests as three principal clinical forms, i.e. cutaneous leishmaniasis (CL), mucocutaneous leishmaniasis (MCL), and visceral leishmaniasis (VL) [1]. CL is the most common clinical presentation and presents as localized disease, which may give rise to more than one primary lesion, satellite lesions, regional lymphadenopathy, and/or nodular lymphangitis [2]. CL, although sometimes self-healing, can cause morbidity and leads to (sometimes severe) mutilation and stigma [3].

In Suriname CL is endemic and a major concern to public health. The disease is generally known in Suriname as ‘bosyaws’ or ‘busi yasi’ - meaning ‘disease from the jungle’ – and is mainly caused by *L.* (*V.*) *guyanensis*, but other infecting species, such as *L. naiffi, L. braziliensis* and *L. amazonensis* are also reported [4, 5]. CL is widespread in the country’s interior, where it mainly affects young males involved in mining, logging and tourism operations, at an annual rate of 6/1000 [6]. Pentamidine isethionate (PI) is the first-line and the only available drug against CL in Suriname, but treatment failures are increasingly reported [5].

The diagnosis of CL is mainly based on a broad variety of clinical signs, but requires laboratory confirmation as these symptoms are not very specific. The laboratory diagnosis of CL is mainly based on microscopic examination of Giemsa’s stained skin scrapings or fine needle aspirates, but this approach is reported to have a low sensitivity [2]. Nucleic acid amplification methods (NAAT), in particular polymerase chain reaction (PCR) or nucleic acid sequence based amplification (NASBA), are reported to have high sensitivity [7], but are difficult to implement in resource limited settings or as (near) point of care (PoC) diagnostics [2, 8].

Recently, two point-of-care (PoC) diagnostic tests have become available that could aid the diagnosis of CL. The first test is a rapid diagnostic test (RDT), the CL Detect™ Rapid Test (InBios International Inc., USA), an immunochromatographic RDT for the detection of the peroxidoxin antigen of *Leishmania* species in CL skin lesions [9]. The second test is a NAAT based on loop mediated isothermal amplification (LAMP) of a conserved region in the *18S rRNA* gene of *Leishmania* species and a specific sequence in the kinetoplast DNA of *L. donovani* [10], which is being marketed as Loopamp™ *Leishmania* Detection kit (Eiken Chemical Co., Japan). It is noted that the Loopamp™ is technically spoken not a true PoC test, as DNA extraction is required, but it ease of performance and the limited infrastructure needed, makes the implementation of this molecular diagnostic almost near patient feasible.

In the present study, the diagnostic performance of these two novel diagnostic tests was determined in comparison to microscopy in a specialized dermatology clinic and PCR (as reference test) performed in a recently established molecular biology laboratory in Suriname.

## Methods

### Study design and population

The study protocol was reviewed and ethically approved by the “Commissie Mensgebonden Wetenschappelijk Onderzoek” of the Ministry of Health of Suriname (date: 29 March 2016, approval VG004a-16).

The recruitment of patients was done between May 2016 and April 2017 and took mainly place at the Dermatology Service of the Ministry of Health (Paramaribo). Some additional patients were recruited whilst visiting the Dermatology outpatient clinical of the Academic Hospital Paramaribo or when encountered by local health staff of the Malaria Program Suriname (Ministry of Health).

All patients with the clinical suspicion of CL were eligible to participate in the study unless: (1) they were less than two years old, (2) did not provide written consent or (3) the required clinical samples needed for the study could not be obtained.

From each CL suspect, two samples from skin lesions were obtained. Sample-1 (skin scraping or) was used to diagnose CL following the routine procedure in Suriname (e.g. smear microscopy). Sample-2, obtained using a dental broach was subjected to three different tests: (1) CL Detect™ Rapid Test, (2) Loopamp™ *Leishmania* Detection Kit and (3) PCR (as reference test).

All CL cases confirmed by routine practice (e.g. microscopy) were given appropriate treatment following the national guidelines.

### Diagnostic procedures

#### Microscopy

Microscopic examination was done on Giemsa stained ulcer smears obtained as per routine practise. The Giemsa-stained slides were examined under light microscope to observe *Leishmania* parasites (amastigotes) and scored as either positive or negative.

#### CL detect™ rapid test

**Fig. 1:**
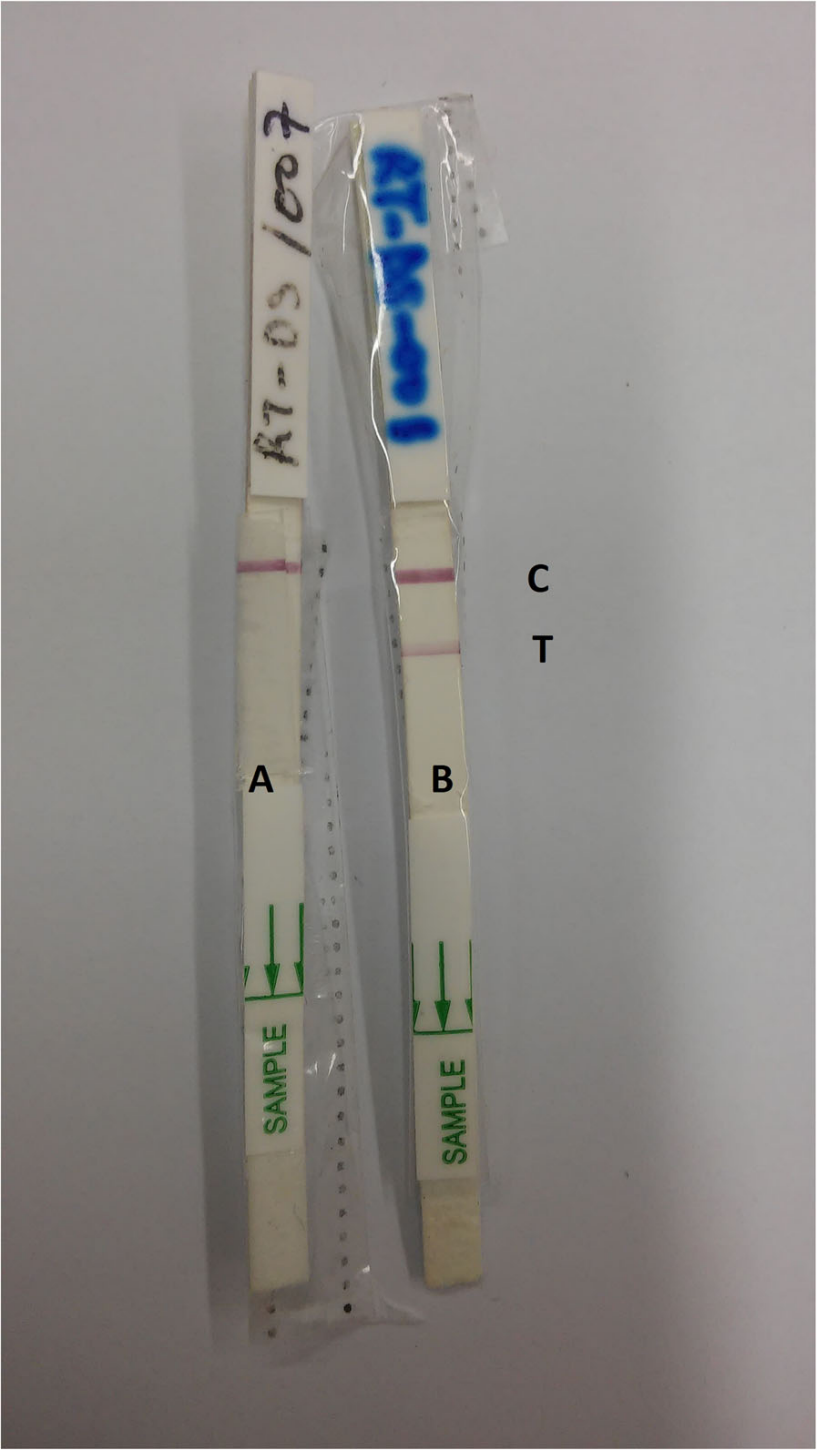
Typical examples of CL Detect™ Rapid Test results. Strip A is negative and strip B is positive. An RDT is considered valid if the internal control line [C] is visible within the recommended reading time of the test. A test is considered positive if also the test-line [T] is visible

A second sample was collected from the same ulcer using a small dental broach, which was subsequently placed according to the instructions of the manufacturer of the RDT in an Eppendorf tube containing 3 drops of lysis buffer (part of the RDT kit) and kept for 25 min at ambient temperature. Twenty microliters of the lysate were transferred to a new Eppendorf tube containing 3 drops of Chase Buffer Type A as provided in the RDT kit. The test strip was inserted in this solution for 20 min before recording the results. A test was considered valid if the internal control line was visible within the recommended reading time of the test (30 min); see Fig. 1 for examples.

The remaining lysate was transported to the Department of Parasitology (Anton de Kom University) and kept at − 20 °C until further PCR and Loopamp testing.

#### DNA extraction

For LAMP and PCR, DNA from the lysate (50 μl) sample was extracted using the QIAamp DNA Mini Kit (QIAgen, Germany) following the manufacturer’s instruction. The DNA was eluted in 100 μl PCR grade water and processed immediately or stored at − 20 °C until further analyses.

Aliquots of DNA (50 μl) were also shipped to the Academic Medical Centre (Amsterdam, The Netherlands) for confirmative LAMP, PCR and *Leishmania* species identification. Confirmative molecular testing was done in The Netherlands to determine the quality of Loopamp, PCR and species identification by RFLP performed in Suriname as these technologies have only recently been introduced in the laboratory in Paramaribo.

#### Loopamp™ *Leishmania* detection kit

**Fig. 2:**
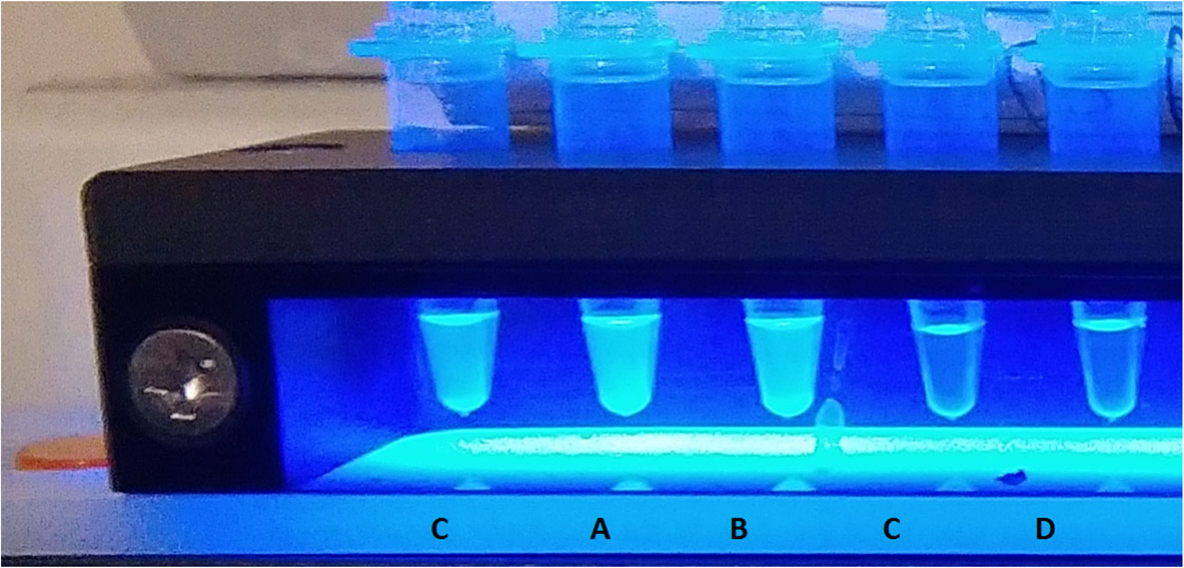
Typical examples of LAMP results. After the LAMP reaction, the samples are illuminated with UV light. *A* positive sample shows turbidity, a negative samples remains clear. *C* positive control sample: *A* and *B* positive samples; *C* and *D* negative samples

Loopamp™ *Leishmania* Detection Kit was performed as per manufacturer’s instructions. Three μl extracted DNA was added to a Loopamp tube plus 27 μl DNA free water. This was run for 40 min at 65 °C and a final step of 80 °C for 2 min in a Loopamp LF-160 incubator in Suriname or a LA-320C in the Netherlands (both from Eiken Chemical Co., Japan). The final result of the Loopamp reaction was visualised via UV illumination and scored as being either positive; see Fig. 2 for examples. Loopamp reactions were performed and interpreted by personnel blinded from the results of the microscopy and CL Detect.

#### PCR and species identification

DNA detection by PCR was performed using 1.25 μl DNA and following the method targeting the *18SrRNA Leishmania* gene for amplification of CL-causing species as described elsewhere [11]. This PCR was used as a reference test to determine the performance of LAMP and RDT.

For species identification, a second PCR targeting the mini exon was performed and amplified PCR products (15 μl) were digested with 10 U restriction enzymes (*Eae* I, *Hae* III and *NCo* I and restriction fragment length polymorphism (RFLP) patterns were compared with those from reference strains *Leishmania (Viannia) guyanensis* (MHOM/BR/75/M4147), *L. mexicana* (MHOM/BZ/82/WR814), *L. amazonensis* (MHOM/SR/2006/SP100), *L. naifi* (MDAS/BR/79/M5533) and *L. braziliensis* (MHOM/BZ/75 M2903) [12, 13].

### Data analysis

Diagnostic performance analysis of the PoC tests under study was done using microscopy or PCR performed in Suriname as reference standards.

Sensitivity and specificity CL Detect and Loopamp were calculated using MEDCALC software (accessible through https://www.medcalc.org/calc/diagnostic_test.php). Confidence intervals for sensitivity and specificity estimates were obtained using the Clopper-Pearson method.

Accordance between the different diagnostic tests was reported in terms of agreement and expressed as kappa-values (Ƙ) using Graphpad Software (accessible through: https://www.graphpad.com/quickcalcs/). Kappa (Ƙ) values (with 95% confidence intervals) report agreement beyond chance and a Ƙ value of 0.60 to 0.80 represented a substantial agreement beyond change, whereas a Ƙ value of > 0.80 represented almost perfect agreement beyond chance.

## Results

### Study population

In total 93 suspected CL cases were enrolled in the present study. The main demographic and clinical characteristics of the patient population is presented in Table 1. The mean age of the study population was 33.6 years, predominantly male (92.5%), of Suriname nationality (80.7%) and the main location of the lesions was on the arm or leg.

**Table 1:** The main demographic and clinical characteristics of the patient population

Age	33,6 years (range: 6–69 years)
Gender [n (%)]	Male: 86 (92.5%)
	Female: 7 (7.5%)
Nationality	Suriname: 75 (80.7%)
	Brazil: 13 (13.9%)
	Others: 5 (5.4%)
Occupation	Mining: 34 (36.6%)
	Logging: 23 (24.7%)
	Agriculture: 6 (6.5%)
	Administrative or domestic service: 12 (12.9%)
	Other: 13 (13.9%)
	No employment: 5 (5.4%)
Main location of the lesion(s)	Head: 7 (7.5%)
	Arm: 31 (33.3%)
	Trunk: 8 (8.6%)
	Leg: 43 (46.2%)
	Arm and Head: 2 (2.2%)
	Leg and Arm: 2 (2.2%)

### Diagnostic performance of the PoC tests

The results of the individual diagnostic tests performed either in Suriname or The Netherlands were as follows. In total 84.9% (79/93) of the suspected CL cases were found positive with microscopy. In contrast, only 33.3% (31/93) were found positive with CL Detect. Molecular testing in Suriname found 80.7% (75/93) cases positive with Loopamp and 87.1% (81/93) with PCR. Confirmative testing in The Netherlands on 92 samples (1 sample could not be analyzed due to the fact that not enough DNA was present in the specimen) revealed 83.7% (77/92) Loopamp positive cases and 84.8% (78/92) PCR positive cases.

There was a very good agreement between PCR testing performed in both countries (k-value: 0.809; 95% CI: 0.627–0.990) and therefore the results of PCR obtained in Suriname were used to determine the diagnostic performance of the PoC tests under study. There was also a good agreement between Loopamp testing performed in Suriname and the Netherlands (k-value: 0.773; 95% CI: 0.600–0.946), indicating that this technology was properly implemented in the laboratory in Suriname.

The diagnostic performance of the PoC tests under study, i.e. CL Detect and Loopamp, is presented in Table 2. It is noted that CL Detect had a very low sensitivity compared to microscopy (36.7, 95% CI: 26.1–48.3%) or PCR (35.8%; 95% CI: 25.2–47.2%)), due to a high number (*N* = 50) of false negative results. The specificity of CL Detect was 85.7% (95% CI: 57.2–98.2%) compared to microscopy and 83.3% (95% CI: 51.6–97.9%) compared to microscopy. In contrast, the Loopamp test had a moderate specificity (42.9, 95% CI: 17.7–71.1%) when compared to microscopy, due to 8 false positive cases, but a good specificity compared to PCR (91.7, 95% CI: 61.5–99.8%). The sensitivity of the Loopamp was 84.% (95% CI: 75.0–91.9%) or 91.4% (95% CI: 83.0–96.5%) compared to microscopy or PCR, respectively.

**Table 2:** Sensitivity and specificity of two point of care diagnostic tests (rapid diagnostic test [CL Detect] or Loop mediated amplification [Loopamp] under evaluation compared to microscopy on Giemsa stained slides (routine procedure) or polymerase chain reaction (PCR) performed in Suriname

Reference test		CL Detect		Loopamp	
		Value	95% CI	Value	95% CI
Microscopy	Sensitivity	36.7%	26.1–48.3%	84.8%	75.0–91.9%
	Specificity	85.7%	57.2–98.2%	42.9%	17.7–71.1%
PCR	Sensitivity	35.8%	25.5–47.2%	91.4%	83.0–96.5%
	Specificity	83.3%	51.6–97.9%	91.7%	61.5–99.8%

### Species identification

Species identification was achieved on 79 of the PCR positive samples in Suriname. All samples, except 2, were typed as *L. guyanensis*. The other 2 samples were typed as *L. amazonensis.* These typing results were confirmed in the Netherlands. In addition, two samples that were found PCR positive in Suriname but could not be typed due to some logistic constraints, i.e. availability of restriction enzymes during the analysis, were identified in the Netherlands as *L. guyanensis.*

## Discussion

In the present study the diagnostic performance of two recently introduced diagnostic tests was evaluated in a study cohort of clinically suspected CL patients in Suriname. The study group was representative for the population that is mainly affected by leishmaniasis in Suriname and comprised mainly of young men infected with *L. guyanensis* [6]. It was noted that 2 cases of infection with *L. amazonensis* were also encountered, which is in line with previous observations suggesting that other species causing leishmaniasis are established in Suriname [4, 5]. This is an important public health concern as there is only one drug available in the country, pentamidine isethionate, which is reported to lose its effectiveness against *L. guyanensis* and might not be effective at all against other *Leishmania* species [5, 14].

The CL Detect performed, in particular in terms of sensitivity, poorly compared to the other diagnostic approaches employed. This was mainly due to the high number of false negative diagnostic results. This result is in contrast to studies reported from Tunisia were an excellent sensitivity (100%) of the test was reported for CL caused by *L. major* [Conference communications by A. Ben Salah et al., ASTMH 2012 Atlanta USA and ASTMH 2014 New Orleans USA cited by [9]. A recent study from Sri Lanka, however, also reported a marked lower sensitivity of the RDT in the diagnosis of CL due to *L. donovani* [9]. In addition, a very recent evaluation in Kabul (Afghanistan), where CL is caused by *L. tropica,* also reported a low sensitivity (65·4%; 95% CI: 59.2–71.2%], but a 100% specificity [95% CI: 80.5–100%] of this RDT [15]. The CL Detect™ Rapid Test was initially developed to detect *L. major,* which often comes at a relative high parasite density, by targeting the peroxidoxin antigen of this parasite. The observed low sensitivity in our study could either be due to the fact that we are studying a New World parasite that might have a lower expression of the peroxidoxin antigen or is producing a different variant of the antigen. The manufacturer describes that the lower detection limit of the test for *L. guyanensis* is between 750 to 2500 parasites and that the peroxidoxin antigen concentration required for a positive result described by the manufacturer is 100 μg/ml [see: http://www.inbios.com/wp-content/uploads/2016/06/900159-00-IVD-CL-Detect-Rapid-Test-Package-Insert.pdf]. It has been established that CL patients in Suriname have a broad range of parasites ranging from just a few per clinical sample to over a million [5]. It might be possible that infections in the lower range were missed by CL Detect, but a quantification of the parasite load was not done in the present study nor was the structure and/or concentration of the peroxidoxin antigen assessed.

Some minor differences have been found in the Loopamp, as well as PCR, results obtained in Suriname and the Netherlands. This is inherent to the fact that these tests have been performed at different locations and by different operators. However, overall the agreement between the molecular test results are (very) good ensuring that the molecular diagnostic tests have been properly performed in the newly established laboratory in Suriname and that these results could be used in the diagnostic comparison.

The Loopamp test demonstrated good diagnostic performance in the present study, which was comparable to a previous study targeting a cohort of Colombian CL suspects [10]. A recent study completed in Afghanistan reported a comparable sensitivity of 87.6% [95% CI: 82.9–91.3%] and a slightly lower specificity of 70.6% [95% CI: 44.0–89.7%] for Loopamp [15]. The LAMP test is targeting a highly conserved region of the *18SrRNA* gene across 8 *Leishmania* species representing both cutaneous (*L. tropica*, *L. major*, *L. braziliensis*, *L. mexicana*, *L. panamensis*, *L. guyanensis*), as well as visceral leishmaniasis (*L. donovani* and *L. infantum)* and covering relevant geographic regions in the Old and the New World [10]. Furthermore, the developed Loopamp test has an analytical limit of detection of around 0.1 parasite per clinical specimen, but is reported to have a slightly lower sensitivity against the two South American strains used during assay development [10]. The diagnostic sensitivity observed in the present evaluation confirms studies that have concluded that molecular tools are more sensitive for the diagnosis of CL [7, 11]. The LAMP showed some reduced specificity (42.9%) when compared to microscopy, and this was caused to 8 “false” positive cases found with the LAMP assay. Molecular tests are in principle much more sensitive that microscopy [2], consequently it is not unexpected that they appear as less specific compared to this test. This is not noted, however, when LAMP is compared to PCR as these two tests have a comparable specificity in the present study.

As Loopamp is simple to perform, does not require expensive equipment and can be used in laboratories with minimal DNA extraction facilities, this diagnostic qualifies to be implemented as a molecular diagnostic test for leishmaniasis in resource limited settings. The Loopamp test is a standardized validated molecular diagnostic test with a proven good performance in terms of sensitivity and specificity [10], in contrast to many in-house developed and applied PCR tests [2].

## Conclusions

The rapid diagnostic test, CL Detect™ Rapid Test, is not suitable for the diagnosis of CL in Suriname. The high sensitivity of Loopamp™ *Leishmania* Detection Kit combined with its ease of use makes it a good candidate for implementation of sensitive molecular diagnosis in resource limited settings. However, in a specialized center, such as the Dermatology Service of the Ministry of Health in Suriname, clinical observation combined with expert microscopy is still sufficient to diagnose CL.
